# Strength Capacity and Failure Mode of Shear Connectors Suitable for Composite Cold Formed Steel Beams: Numerical Study

**DOI:** 10.3390/ma14133627

**Published:** 2021-06-29

**Authors:** Sherif A. Elsawaf, Saleh O. Bamaga

**Affiliations:** 1 Department of Civil Engineering, College of Engineering, University of Bisha, Bisha 61922, Saudi Arabia; selsawaf@ub.edu.sa; 2 Department of Structural Engineering, Faculty of Engineering, Al Azhar University, Cairo 11371, Egypt

**Keywords:** composite beam, CFS section, push test, shear connector, finite element modeling

## Abstract

In this paper, the findings of numerical modeling of the composite action between normal concrete and Cold-Formed Steel (CFS) beams are presented. To obtain comprehensive structural behavior, the numerical model was designed using 3-D brick components. The simulation results were correlated to the experimental results of eight push tests, using three types of innovative shear connectors in addition to standard headed stud shear connectors, with two different thicknesses of a CFS channel beam. The proposed numerical model was found to be capable of simulating the failure mode of the push test as well as the behavior of shear connectors in order to provide composite action between the cold-formed steel beam and concrete using the concrete damaged plasticity model.

## 1. Introduction

Currently, there is extensive use for Cold-Formed Steel (CFS) sections in the construction industry. They are used as lightweight load-bearing frames [1]. In the context of of its widespread use, the first standard code for CFS construction was published in 1946 [2]. To improve CFS sections’ strength and toughness and prevent instability due to buckling, stiffening CFS sections by edge stiffeners was introduced [3,4]. In residential building construction, the use of CFS sections has become increasingly popular, due to their appreciable efficiency [5]. It is a relatively new concept to develop composite action between CFS sections and concrete to form composite beams in small and medium buildings [6,7,8,9]. The difficulty of introducing conventional shear connectors to CFS sections due to a lack of specifications may be the reason behind the limitations of its usefulness in composite applications.

However, researchers were excited to conduct investigations on composite CFS concrete members. For example, an innovative lightweight composite bridge girder was investigated [10]. A composite concrete CFS slab connected using a stand-off screw was studied by [11]. Composite concrete slabs with CFS I-sections and new CFS shear connectors connected by self-drilling screws and welding were investigated by [12]. Composite concrete CFS beams and slab joists with different bent-up, pre-drilled holes, self-drilling screws, a CFS channel, and angle shear connectors were experimentally studied by Irwan et al. [13,14,15]. Lakkavalli and Liu [16], and Malite et al. [17]. In composite concrete steel slabs, parallel or perpendicular metal decking is usually used. Therefore, developing shear connectors suitable for composite metal decking concrete CFS floors is recommended [18]. The important part of such composite concrete CFS beams is the shear connectors that are essentially responsible for an efficient composite system [19]. Experimental investigations were conducted on composite metal decking concrete beam systems with CFS sections [20,21]. However, so far, this new topic needs more investigation to fill a knowledge gap. Therefore, more experimental and numerical works are highly recommended [22]. Due to the higher cost of experimental works that make it difficult to provide sufficient information, numerical modeling has become an alternative reliable approach. Several researchers have used this approach [19,23,24].

## 2. The Proposed Shear Connectors and Push Test Setup

**Figure 1:**
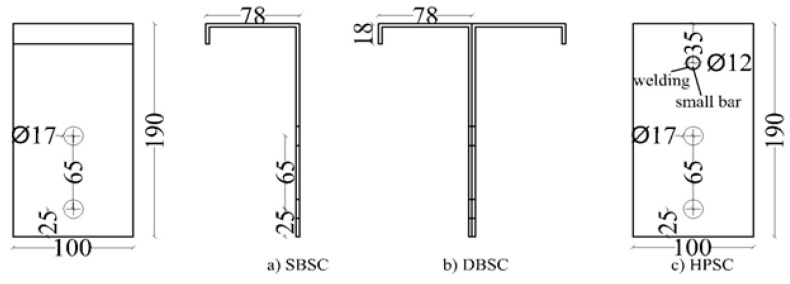
Types of shear connectors: (**a**) SBSC shear connector, (**b**) DBSC shear connector, (**c**) HPSC shear connector [21].

**Figure 2:**
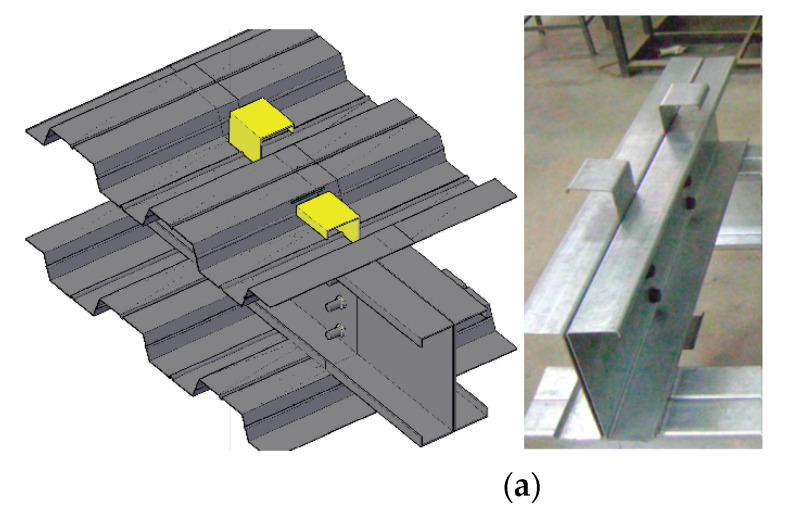

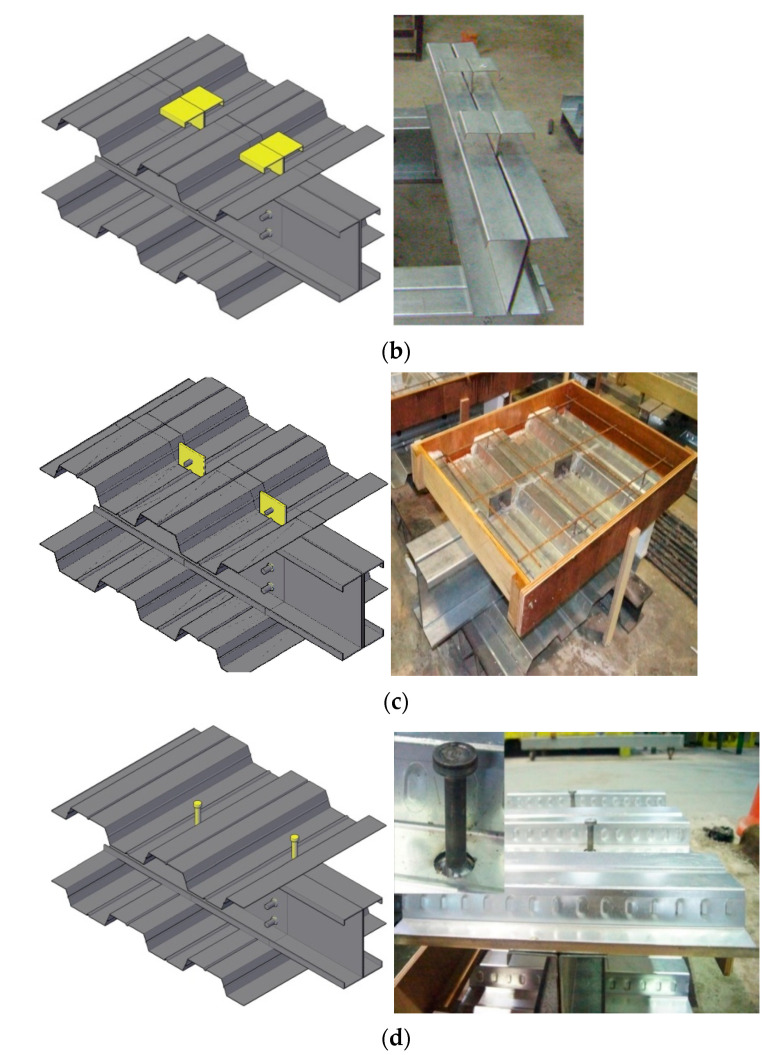
Shear connectors’ arrangement [21]. (**a**) SBSC shear connector. (**b**) DBSC shear connector. (**c**) HPSC shear connector. (**d**) HSSC shear connector.

Innovative composite concrete CFS beams were studied. Two CFS sections with different thicknesses (i.e., 2.00 mm and 2.3 mm) were used. Three innovative shear connectors made of CFS sections were investigated. They are namely SBSC (Figure 1a), DBSC (Figure 1b), and HPSC (Figure 1c). The details of the proposed composite beams and shear connectors are presented in [21]. Moreover, the conventional headed stud shear connectors, namely HSSC, were also investigated. A Headed stud shear connector (HSSC) of 16 mm in diameter, a 76 mm height, and with the yield strength of 450 MPa was used to investigate the feasibility and behavior of welded studs to a thin flange of a CFS section. The stud was welded to the top flange of the steel beam. The welding was formed from the top and bottom faces of the steel flange. The top flange of the steel beam was pre-holed using a 16 mm diameter to allow for such a welding method. Gas metal arc welding with electricity conditions of 20 A and 68 V was used to perform the welding around the stud connector from both sides. Figure 2 depicts the arrangement of the proposed shear connectors in push test specimens.

**Figure 3:**
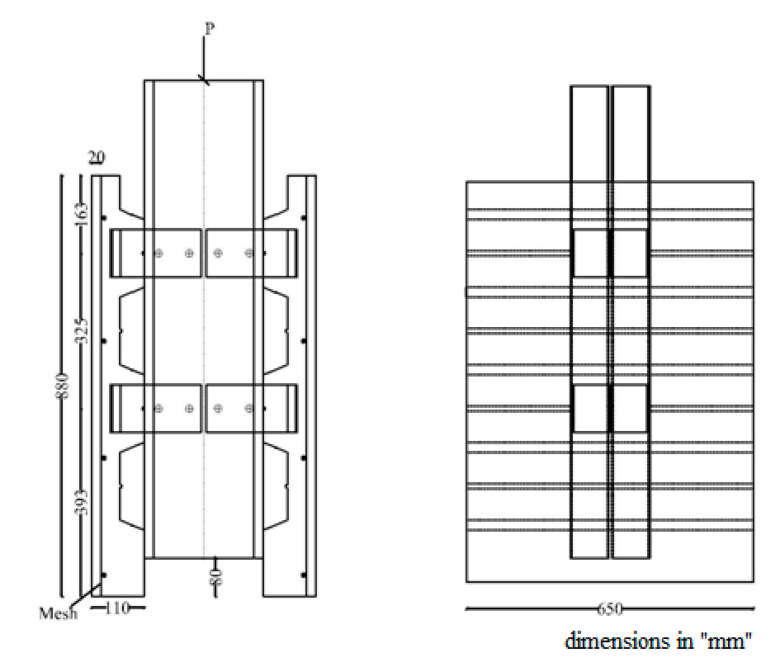
Test configuration for a DBSC250 specimen [21].

**Figure 4:**
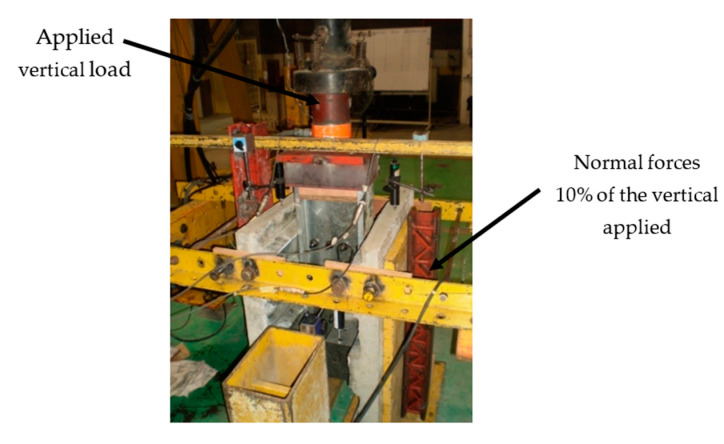
Push test set up [21].

To allow for slippage during the test, all specimens were constructed with an 0.080 m reset between the ends of the concrete slab and the steel beam. The schematic push test specimen is shown in Figure 3. The push test setup is shown in Figure 4. The test was stopped when the specimen could no longer handle the additional load, and the overall load was decreased by 20%. 

## 3. Description of the FE Model 

**Figure 5:**
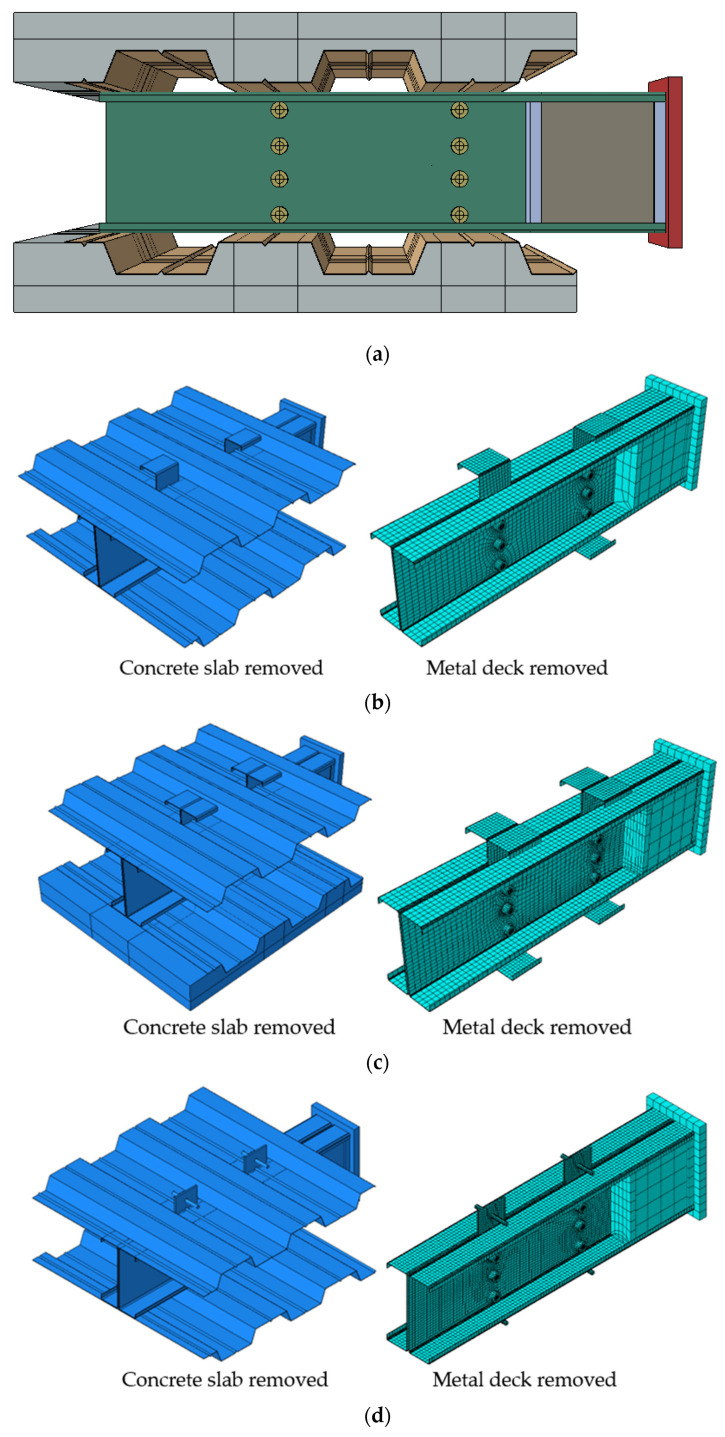

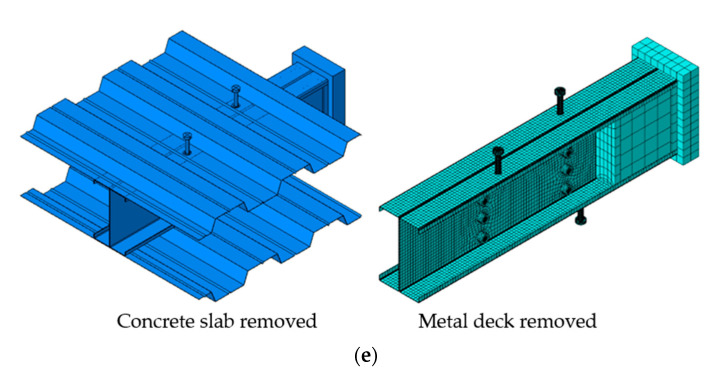
Finite element models of push tests. (**a**) Overall model; (**b**) Single Bracket Shear Connector (SBSC); (**c**) Double Bracket Shear Connector (DBSC); (**d**) Hot Rolled Plate Shear Connector (HPSC); (**e**) Standard headed Shear Connector (HSSC).

We conducted 3-D nonlinear FE simulations using ABAQUS software [25], while considering the material and geometric nonlinearities. The geometric nonlinearity was considered by activating “NLGEOM” in ABAQUS/Standard. This was done to account for significant deformation and local instability results. Figure 5 shows the finite element model for the structural arrangement of the push test for the proposed shear connectors to be simulated in this research. 

The simulation methodology can be summarized as follows:

- Three-dimensional solid elements (C3D8) were used to model the key structural members to obtain detailed structural behavior (CFS beam, bolts, concrete slab, and shear connector components).
- Mechanical properties of the steel beams and shear connectors [20,21] were obtained in compliance with BS EN ISO 6892-1:2009. These properties were used to develop the stress-strain constitutive relationships used in the FE models. Table 1 shows the yield strength, ultimate strength, and elastic modulus.

- For reinforced concrete elements, the ABAQUS program’s concrete damaged plasticity model for reinforced concrete elements was used, which can reflect the complete inelastic behavior of concrete in stress and compression, including damage characteristics. The concrete damaged plasticity model, which takes into account isotropic elastic damage and plastic behavior of materials, can simulate tensile cracking and compressive crushing of concrete materials [26,27].
- For bolts and nuts, the mechanical properties were assumed to be elastic-perfectly plastic. For Grade 8.8 bolts, the yield strength was 640 MPa and the elastic modulus was 210,000 MPa.
- The welding between headed stud shear connectors and CFS flange were modeled using the ‘‘tie’’ type constraint in ABAQUS.
- To prevent the FE model to move or twist, it was restrained from both sides to simulate the actual boundary conditions, as shown in Figure 3 and Figure 4.
- The ABAQUS contact function was used to model the interaction between components, including the interface between the two channels, between the concrete slab and the metal deck, between the metal deck and the beam, between the shear connectors and concrete slab, between the bolt shanks and the web of the beam, between the bolt heads and the web of the beam, and between the web of the channel and the shear connectors.
- A contact was simulated as a surface-to-surface contact with a small sliding choice. To avoid mutual penetration of the steel and concrete in the normal direction, a “hard contact” was assumed for the normal contact behavior, while a friction contact with a coefficient of m = 0.3 was applied tangentially to the surface of the contact pairs.
- The loads were applied to the beam at one-point loads (as shown in Figure 3 and Figure 4) and were increased gradually until failure. In addition, normal forces were sustained for both concrete slabs using a yoke assembly operated by a hand hydraulic pump to replicate the actual situation of the composite beam in structures and prevent the rotation of the last studded rib at the top of the push test specimen. The magnitude of the normal force is kept at about 0.1 of the vertical applied loads.
- A basic Rankine criterion was used to detect crack initiation for crack detection [28]. It was also assumed that cracking occurs when the tensile equivalent plastic strain exceeds zero and the maximum principal plastic strain also exceeds zero [29].
- The values of the damage parameters used in the model are as follows: dilation angle = 30, eccentricity e = 0.1, the ratio of initial equibiaxial compressive yield stress to initial uniaxial compressive yield stress was fb0/fc0 = 1.16, the ratio of the second stress invariant on the tensile meridian to that on the compressive meridian Kc = 0.6667, viscosity parameter = 0.0001.

## 4. Results and Discussion

The experimental push tests conducted by [20,21] were simulated by the authors using the ABAQUS software package. In this investigation, the FE results are presented in main two groups: deformed shapes of each component and the modes of failure.

### 4.1. Bracket Shear Connectors

**Figure 6:**
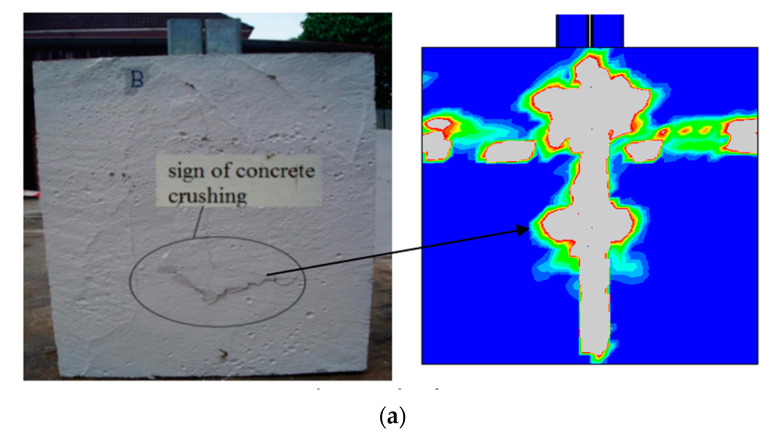

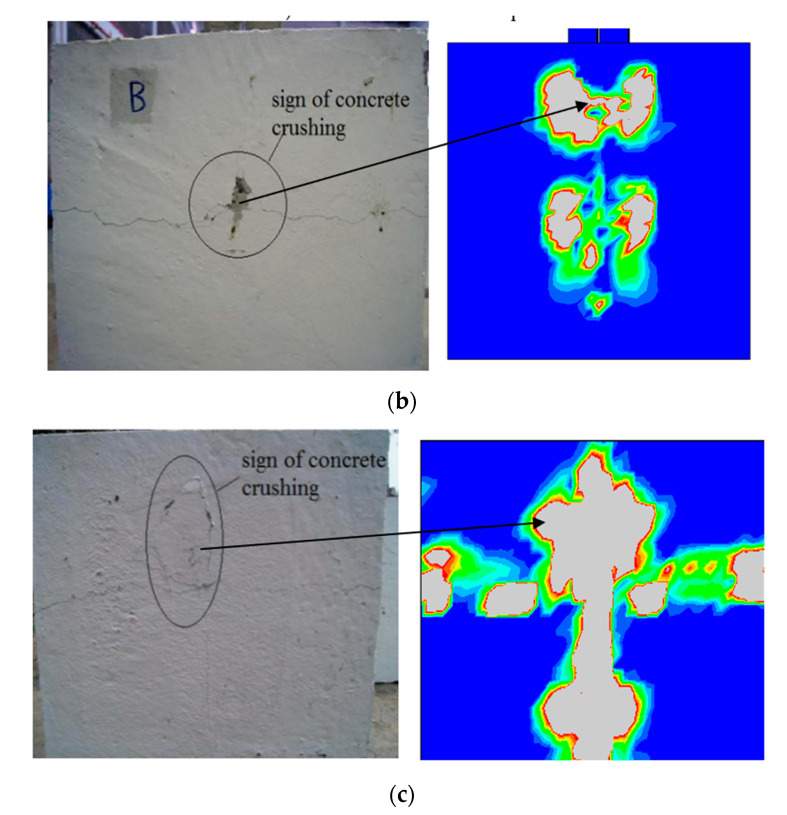
Failure modes of push test specimens compared to proposed FE model. (**a**) Failure mode of SBSC specimen. (**b**) Failure mode of DBSC specimen. (**c**) Failure mode of HPSC specimen.

The observed failure modes of SBSC, DBSC, and HPSC push test specimens are similar. These types of shear connectors failed by concrete crushing proved by cracks observed along the slabs located in the center of slabs, which is captured by the finite element results as shown in Figure 6, which represent the pattern of the typical cracks after failure for the SBSC, DBSC, and HPSC. As can be seen in Figure 6, an excessive plastic strain which is represented by a transverse crack occurred underneath the concrete rib of the top and bottom level of shear connectors. The reason for these longitudinal cracks is due to the fact that as applied load increases, the longitudinal shear force transferring from concrete slab to steel beam through shear connectors tends to split the concrete slab longitudinally.

**Figure 7:**
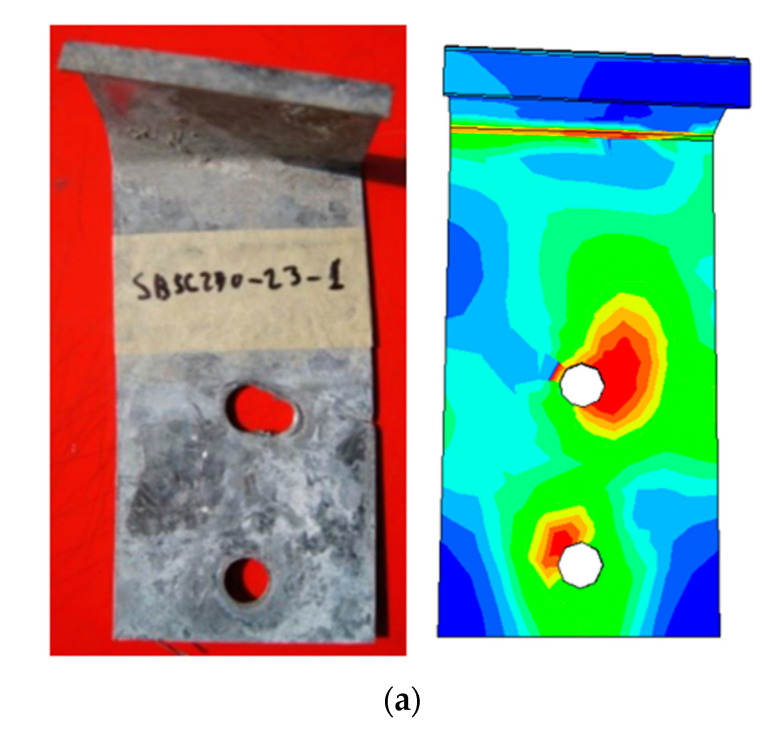

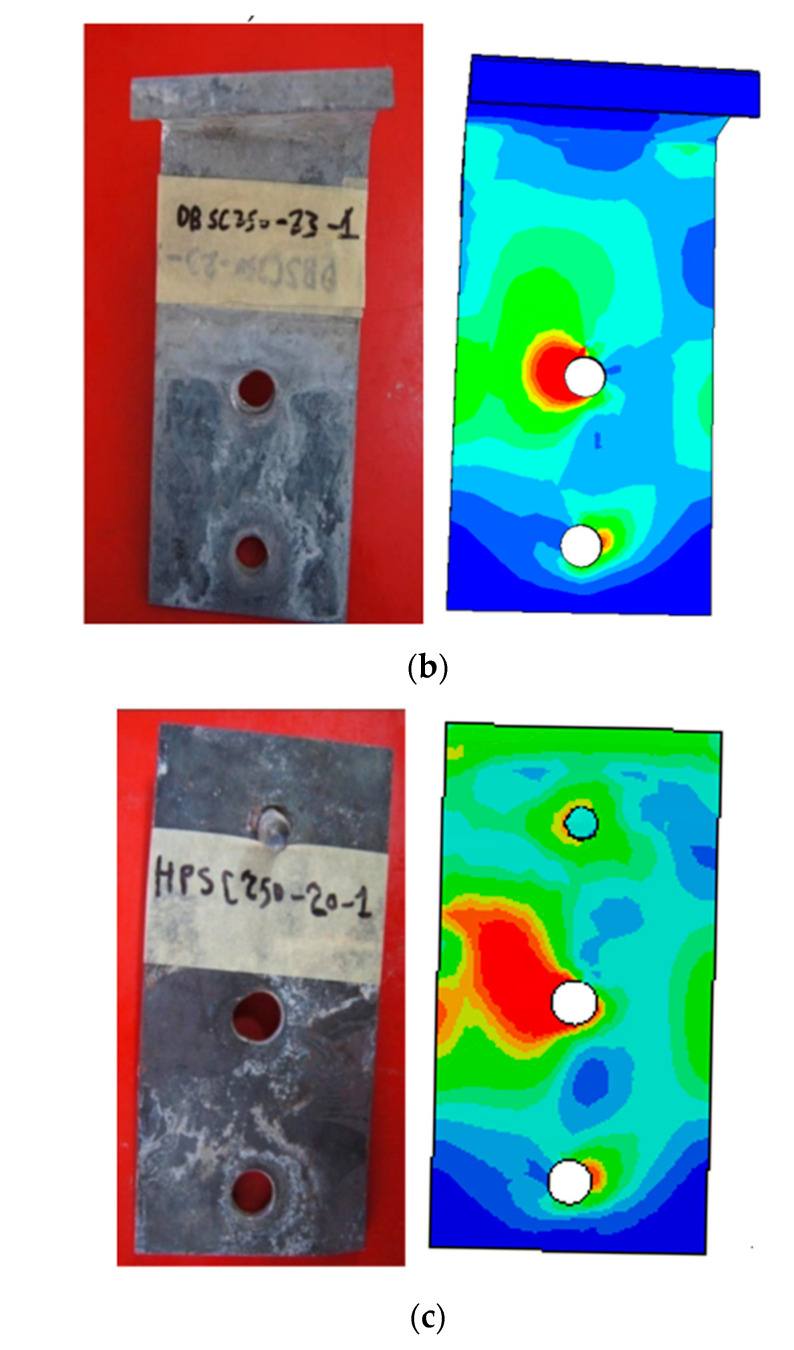
Comparison of deformed shear connectors from the FE model and push test specimens. (**a**) SBSC Shear Connector. (**b**) DBSC Shear Connector. (**c**) HPSC Shear Connector.

In the test, it was found that SBSC, DBSC, and HPSC shear connectors were rotated (a very small rotation). As a consequence of the initial rotation of SBSC and DBSC shear connectors, the shear connector was deformed, as can be seen from Figure 7a,b. For HPSC shear connectors, no deformation on the part of the shear connector embedded in the concrete slab was observed, as can be seen from Figure 7c. 

Figure 7 shows a concentration of stresses around the bolt hole near the CFS beam flange, indicating a strong bearing around the bolt of shear connector near the concrete slab in the test results for SBSC and HPSC specimens, and a low degree of bearing for DBSC specimens. Since the shear connector was able to move around the bolt (i.e., far from the concrete slab) after the concrete was crushed, the bolt near the concrete slab was the only one that could resist the applied load, causing bolt bearing failure.

**Figure 8:**
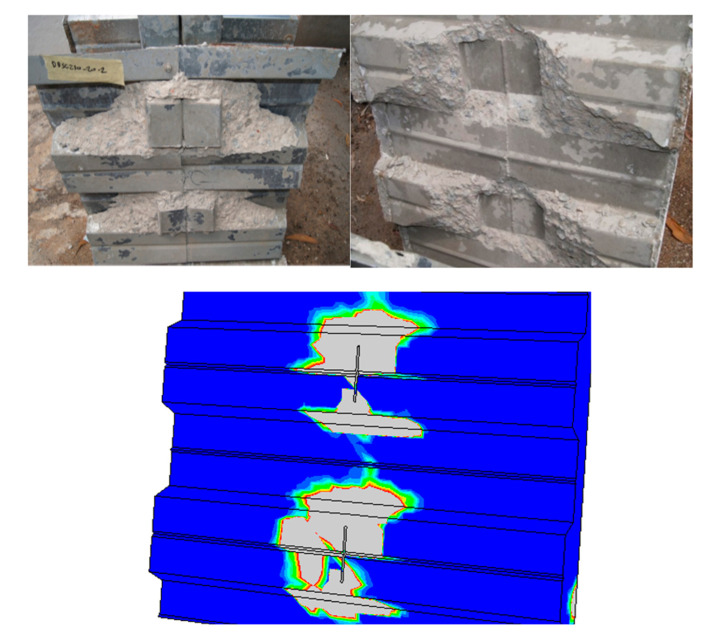
Failure mode of the DBSC test specimen compared to the proposed FE model.

In DBSC specimens, Figure 8 indicates an excessive separation between both the concrete slab and the metal deck. This is due to the DBSC shear connector’s wide flange surface (double of the SBSC), which results in a weak connection between the concrete’s upper and lower surfaces.

### 4.2. Standard Headed Stud Shear Connector (HSSC)

**Figure 9:**
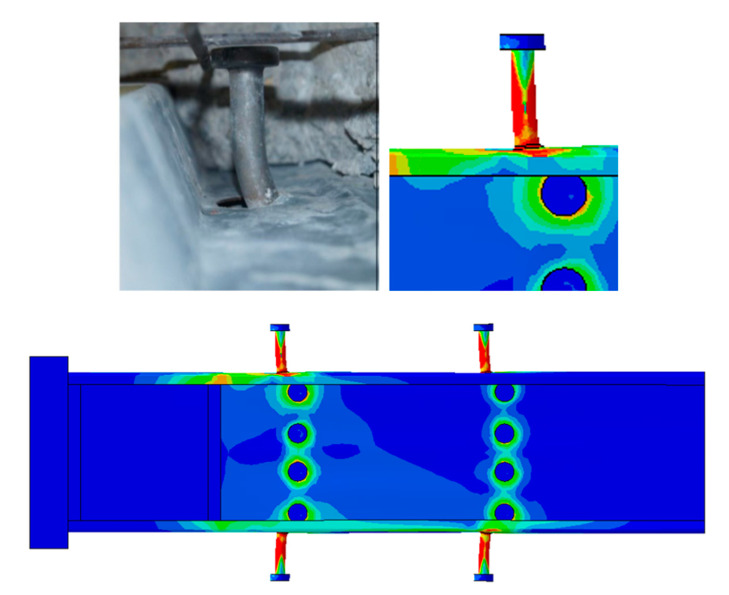
Failure mode of the HSSC test specimen compared to the FE model.

**Figure 10:**
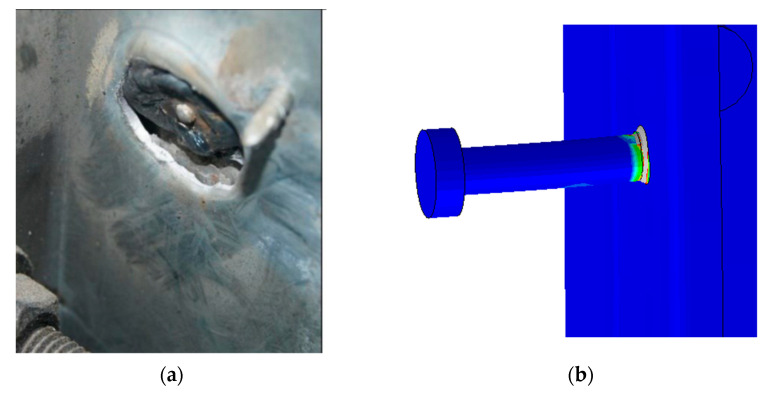
Plastic strain distribution from the FE model and pulling-out of the stud from test specimen. (**a**) Push test specimen. (**b**) Plasic strain in the FE model.

For push test specimens with headed stud shear connectors (HSSC), the observed failure mode was caused by deformation of the shank of the stud followed by pulling-out from the thinner flange of steel beam, as shown in Figure 9. This mode of failure was captured by the finite element by the excessive plastic strain in weld connected the stud to the thinner flange of steel beam, as shown in Figure 10. No cracks at the concrete slabs, as well as no separation between the concrete slab and metal deck, were observed. 

## 5. Validation of the Proposed FE Model 

Table 2 shows the strength capacity of the shear connectors based on experimental and finite element results. For the SBSC, DBSC, HPSC, and HSSC shear connectors, a very good agreement between experimental and finite element results was observed, ranging from 0.94 to 1.04. With such a clear correlation, it was possible to conclude that the FE model accurately depicts the behavior and strength capacity of the proposed composite beams with the CFS section.

## 6. Conclusions

This study was focused on developing an FE model to simulate the composite CFS beam behavior. A total of eight push tests were modeled using non-linear material properties of concrete and steel and concrete damaged plasticity model in ABAQUS. Four types of shear connectors were modeled (namely SBSC, DBSC, HPSC, and HSSC shear connectors) with two different thicknesses of CFS channel beam. The following conclusions may be drawn:

- The developed finite element models are in good agreement with the experimental results of the push tests and observations. The deformed shapes and the relevant failure modes were accurately captured by the model for all types of shear connectors. For example, the concrete crushing and shear connector rotation in SBSC, DBSC, and HPSC push test specimens were clearly captured by the FE model.
- The concrete damaged plasticity model in ABAQUS can accurately capture crushing and the longitudinal crack of a concrete slab, which is the common failure mode for SBSC, DBSC, and HPSC.
- The developed finite element models accurately model the interaction between the metal decking and concrete slab. For DSBC, the separation between the concrete slab and metal deck occurred is clearly captured by the proposed finite element models. This weak connection is due to the DBSC shear connector’s wide flange surface (double of the SBSC) that leads to such separation.
- The bearing around the bolt of shear connector that is near to the concrete slab (which was the only bolt resisting the rotation after concrete slab crushing) in the test results of SBSC, DBSC, and HPSC specimens is accurately predicted by the model by observed stress concentration around the bolt hole connecting the shear studs to the web of the cold formed beam.
- The proposed finite element models have clearly captured the failure modes of HSSC specimens being the deformation of the shank and the pulling-out of the stud from the thinner flange of steel beam for HSSC specimens, while no crack was observed in the concrete slab.
- The finite element results of ultimate loads were found to be in very acceptable agreement with experimental data, ranging between 0.94 and 1.04. However, the results of the section analysis are generally conservative compared to experiments.

## Figures and Tables

**Table 1:** Materials Properties.

Specimen	F_y_ (MPa)	F_u_ (MPa)	F_u_/F_y_	E (GPa)	Elongation (%)
SC250-23	518.67	558.45	1.07	187.42	9.20
SC250-20	542.65	575.74	1.06	191.05	9.10
Hot rolled plate	321.79	464.82	1.44	185.6	23.70
Reinforcement mesh	640.82	676.91	1.05	191.67	-
SDP51-10	678.37	687.00	1.01	201.25	-

F_y_: yield tensile strength of steel; F_u_: ultimate tensile strength of steel; E: Young’s modulus.

**Table 2:** Experimental and FE results comparison.

Specimen	Avg. *P*_u,per connector_ (kN)	Predicted Value by Finite Element Results	
		*P*_pre. Per connector_ (kN)	Averg. Exp./pre. Ratio
SBSC250-20	43.03	43.63	0.99
DBSC250-20	52.15	55.34	0.94
HPSC250-20	55.45	55.27	1.00
HSSC250-20	54.93	53.80	1.02
SBSC250-23	44.58	43.00	1.04
DBSC250-23	53.60	55.79	0.96
HPSC250-23	55.15	55.45	0.99
HSSC250-23	53.97	53.80	0.94

## Data Availability

Not applicable.
